# Young T Cells Age During a Redirected Anti-Tumor Attack: Chimeric Antigen Receptor-Provided Dual Costimulation is Half the Battle

**DOI:** 10.3389/fimmu.2013.00135

**Published:** 2013-06-05

**Authors:** Andreas A. Hombach, Hinrich Abken

**Affiliations:** ^1^Center for Molecular Medicine Cologne, University of Cologne, Cologne, Germany; ^2^Department I Internal Medicine, University Hospital Cologne, Cologne, Germany

**Keywords:** adoptive cell therapy, chimeric antigen receptor, memory T cells, CD28, OX40

## Abstract

Adoptive therapy with chimeric antigen receptor (CAR)-redirected T cells showed spectacular efficacy in the treatment of leukemia in recent early phase trials. Patient’s T cells were *ex vivo* genetically engineered with a CAR, amplified and re-administered to the patient. While T cells mediating the primary response were predominantly of young effector and central memory phenotype, repetitive antigen engagement irreversible triggers T cell maturation leaving late memory cells with the KLRG1^+^ CD57^+^ CD7^−^ CCR7^−^ phenotype in the long-term. These cells preferentially accumulate in the periphery, are hypo-responsive upon TCR engagement and prone to activation-induced cell death. A recent report indicates that those T cells can be rescued by CAR provided CD28 and OX40 (CD134) stimulation. We discuss the strategy with respect to prolong the anti-tumor response and to improve the over-all efficacy of adoptive cell therapy.

## Adoptive Therapy with CAR Engineered T Cells Showed Spectacular Efficacy in Early Phase Trials

Recent success in the immune therapy of malignant diseases has sustained the promise that the immune system can control cancer in the long-term. On the other hand, tumor-specific T cells are rare in cancer patients making their isolation and *ex vivo* amplification to therapeutic numbers necessary. To overcome the situation strategies were developed to engraft T cells with defined specificity by genetic engineering; the so-called “T-body” strategy equips patient’s T cells with a recombinant trans-membrane receptor molecule which is composed in the extracellular part of an antibody-type recognition domain for MHC-independent binding and in the intracellular part of T cell activating domains, mostly the TCR CD3ζ endodomain linked to a costimulatory domain like CD28, OX40, or 4-1BB (Gross and Eshhar, 1992; Eshhar, 2008). Such a chimeric antigen receptor (CAR) redirects T cells in an antigen-specific fashion producing specific T cell activation toward defined targets.

Second generation CAR’s providing CD28 costimulation along with the primary CD3ζ signal more effectively activate T cells than CAR’s with CD3ζ signaling only. This is basically due to the CD28 mediated improvement of T cell effector functions and the protection from activation-induced cell death resulting in prolonged persistence *in vivo* (Savoldo et al., 2011). Other costimulatory domains of the CD28 family, like 4-1BB (CD137), also improves T cell persistence (Milone et al., 2009; Song et al., 2011). Each individual costimulatory signal, however, differentially orchestrates the effector functions including cytokine secretion, amplification, and cytolytic activity (Hombach and Abken, 2011) which allows to modulate the anti-tumor response in a fine-tuned fashion.

Current clinical trials are utilizing second generation CARs to ensure prolonged persistence of engineered T cells *in vivo*. T cells engineered with a 4-1BB-ζ CAR targeting CD19 recently produced spectacular efficacy toward refractory leukemia in patients with high tumor burden (Kalos et al., 2011; Grupp et al., 2013). Further studies in other centers are also reporting encouraging clinical responses using CD28-ζ CAR T cells (Brentjens et al., 2011; Kochenderfer et al., 2012). The general success of these studies is likely due to repetitive tumor cell killing by CAR T cells; additionally, the targeted CD19 is also expressed by healthy B cells which re-stimulate the CAR T cells independently of the targeted tumor cells. The situation in targeting solid tumors, however, is more complex, in particular with respect to immune repression, and may require supporting strategies as discussed in a recent review (Gilham et al., 2012). We here address an additional aspect which is attracting increasing attention and which is of equal relevance for the success in adoptive cell therapy: the progression of CAR redirected T cells into terminal maturation upon repetitive antigen encounter.

## Repetitive CAR Engagement Produces Late Effector Memory Cells with Altered Functional Capacities

**Figure 1:**
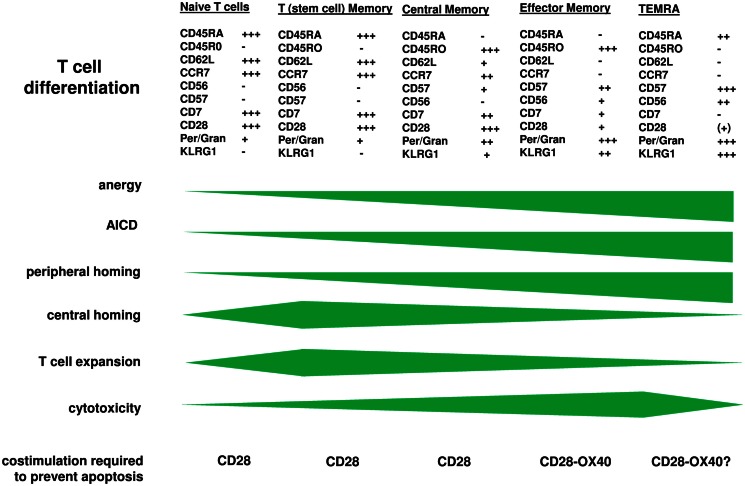
**T cell maturation is accompanied by altered functional properties**. CAR engineered T cells progress in maturation when the CAR repetitively engages cognate antigen as physiologically occurs upon TCR/CD28 signaling. While CAR engineered “young” memory T cells are transferred to the patient, the cells expand and undergo further differentiation leaving effector memory T cells at the tumor lesion which require additional signals for being protected from anergy and activation-induced cell death. On the other hand, those more matured cells have increased cytolytic potential making them highly effective in the anti-tumor attack. Engineering T cells with a combined CD28-OX40 CAR which prevents apoptosis of matured CCR7^−^ T cells is one of the upcoming strategies to solve the situation and to improve the anti-tumor efficacy in the long-term.

Chimeric antigen receptor engineered young T cells, the majority of them with central memory phenotype, are adoptively transferred to the patient since these cells showed superior in mediating an anti-tumor response in pre-clinical models (Klebanoff et al., 2005). Repetitive binding to cognate antigen, however, induces CAR T cells to amplify, as T cells physiologically do upon TCR/CD28 engagement or TCR stimulation in presence of IL-2. Extensive amplification, however, substantially impacts the anti-tumor efficacy of CAR T cells in the long-term. After <2 weeks of cell divisions *in vitro*, T cells progress in maturation and alter their functional properties, associated by a change in phenotype (Figure 1). Repetitive antigen engagement converts naïve and central memory T cells to cells with a CCR7^−^ CD62L^low^ CD57^+^ KLRG1^+^ effector memory phenotype with CD45RO^high^ CD45RA^low^ and CD27^low^ CD28^low^ expression. CAR mediated maturation occurs in both CD8^+^ and CD4^+^ T cells and does not happen when cell division is blocked. The process is observed in a mouse tumor model in which after adoptive transfer of young CCR7^+^ CAR T cells the majority of tumor infiltrating CAR T cells are of CCR7^low^ phenotype (Hombach et al., 2013). One consequence is that the capacity of those cells to re-enter the lymph and to re-circulate is diminished since CCR7 is required for T cell homing into secondary lymphoid organs (Sallusto et al., 1999; Müller and Lipp, 2003; Bromley et al., 2005; Klebanoff et al., 2005; Moschovakis and Förster, 2012). Inability of CCR7^−^ T cells to re-circulate, on the other hand, may be of advantage since most solid cancer lesions occur in the periphery. The assumption is sustained by the observation that CCR7^−^ CAR T cells persist in higher numbers in the tumor lesion although both the CCR7^+^ and CCR7^−^ subset T cells equally efficiently target to the tumor (Hombach et al., 2013). Paradoxically, the anti-tumor response of CCR7^−^ CAR T cells is less efficient than that of CCR7^+^ T cells when redirected by a CD28-ζ CAR. This is moreover unexpected since CCR7^−^ T cells secrete higher amounts of pro-inflammatory cytokines like IFN-γ and harbor higher levels of cytolytic effector molecules like perforin and granzymes compared to CCR7^+^ T cells. Detailed analyses revealed that CCR7^−^ T cells are prone to spontaneous and activation-induced cell death which is insufficiently prevented by CAR mediated CD28 costimulation (Hombach et al., 2013). Similar observations were reported for CD57^+^ T cells (Chattopadhyay et al., 2009) and which is in contrast to CCR7^+^ T cells.

Simultaneous CD28 and OX40 costimulation reduces the high propensity of CCR7^−^ T cells to undergo apoptosis (Hombach et al., 2013). OX40 promotes Bcl-xL and Bcl-2 expression, enhances the survival of antigen-experienced effector T cells and improves the generation of antigen-specific T cell memory (Rogers et al., 2001). In combination with CD28 in a so-called “third generation” CD28-OX40 CAR, combined costimulation improved survival and cytolytic activities of CCR7^−^ T cells toward targeted cancer cells (Hombach et al., 2013). Similar results were obtained with CD62L^−^ T cells which mature from CCR7^−^ CD62L^+^ T cells upon stimulation (Hombach and Abken, 2011). The combination of costimulatory signals achieves the effect since OX40 alone does not provide benefit in this context whereas CD28 costimulation alone, which prevents CCR7^+^ T cell apoptosis, does not reduce the number of apoptotic CCR7^−^ cells. Taken together adoptive cell therapy will benefit from redirecting T cells by a CD28-OX40 CAR to provide protection from apoptosis when young cells progress in maturation.

## Perspectives: How to Maintain an Anti-Tumor Response in the Long-Term?

Several factors together are required to rescue matured CAR T cells for the anti-tumor response in the long-term.

First, any T cell subset converts to effector memory cells upon repetitive antigen engagement and amplification and in each stage of maturation requires appropriate costimulation to escape cell death; consequently, harnessing young T cells with a CD28-OX40 CAR will be beneficial and will rescue CCR7^−^ cells when produced during an anti-tumor attack.

Second, CCR7^−^ T cells persist in peripheral lesions due to their inability to re-enter the lymph, thereby increasing the probability for successful cancer cell killing. To improve their accumulation at the tumor site T cells were additionally engineered with the CCR2 receptor (Moon et al., 2011). Alternatively, CAR engineered T cells are injected into the tumor lesion taking advantage of the plethora of different effector T cell subsets in fighting cancer while circumventing the limitations in T cell trafficking. In contrast, i.v. injected T cells become stuck in the lungs for hours and subsequently accumulate in liver, spleen, and lymph nodes, while regional application produces T cell persistence at the injected tumor site with only local diffusion within the following days (Parente-Pereira et al., 2011). The strategy, however, requires good accessibility by direct puncture or by endoscopy and is currently evaluated in the treatment of head and neck cancer (EudraCT 2012-001654-25, NCT01722149) or will be applied to the treatment of cutaneous lymphoma (EudraCT 2011-003125-10).

Third, effector memory T cells produce increased levels of pro-inflammatory cytokines and cytolytic molecules, both contributing to improve cancer cell killing. Late memory T cells, however, are TCR hypo-responsive which is due to an inefficient formation of the TCR synapse as a result of galectin-3 anchoring of TCR components. Interestingly, formation of a transgenic CAR synapse in those cells is not affected making them fully responsive to CAR targets (Rappl et al., 2012).

Since T cell expansion is mandatory to establish adoptively transferred T cells in the long-term, sufficient space is provided to transferred T cells by non-myeloablative lympho-depleting pre-conditioning followed by IL-2 administration to sustain expansion; other cytokines like IL-7 and IL-15 are also explored (Weber et al., 2011). Extensive T cell amplification, on the other hand, produces effector memory T cells which then need to be protected from activation-induced cell death. Other costimulation than by CD28 and OX40 may also provide benefit to those cells, for instance 4-1BB (Song et al., 2011). Co-signaling by 4-1BB and CD28 may also provide an advantage to matured T cells, however, needs to be evaluated in detail. The effect of each combination of costimulatory signals, however, cannot be predicted since the CAR with its linked endodomains provides simultaneous signaling while in the physiological situation the signals occur individually in a well-defined temporal and spatial order. An elegant solution of the dilemma is the use of virus-specific T cells which are further matured by the immune system and have some significant properties needed for effective anti-cancer therapy. These cells obtain survival and costimulatory signals when engaging virus-infected cells by their TCR. Current trials use EBV or CMV specific, autologous T cells engineered with a first or second generation CAR, for instance directed against ErbB2 (NCT01109095), CD30 (NCT01192464), CD19 (NCT00709033; NCT01475058; NCT01430390; NCT00840853; NCT01195480), or GD-2 (NCT00085930). Virus-specific T cells are long-lived in pre-clinical models *in vivo*, have a great capacity to amplify and are particularly applied in the context of allogeneic stem cell transplantation where they protect from virus re-activation and tumor relapse while having low risk of inducing graft versus host disease.

## Conflict of Interest Statement

The authors declare that the research was conducted in the absence of any commercial or financial relationships that could be construed as a potential conflict of interest.
